# Genetic insights into the effect of trace elements on cardiovascular diseases: multi-omics Mendelian randomization combined with linkage disequilibrium score regression analysis

**DOI:** 10.3389/fimmu.2024.1459465

**Published:** 2024-12-03

**Authors:** Bohang Chen, Chuqiao Wang, Wenjie Li

**Affiliations:** ^1^ The First Clinical Medical College, Liaoning University of Traditional Chinese Medicine, Shenyang, Liaoning, China; ^2^ The Department of Endocrinology, Liaoning Health Industry Group Fukuang General Hospital, Fushun, Liaoning, China; ^3^ The Department of Cardiovascular Medicine, Affiliated Hospital of Liaoning University of Traditional Chinese Medicine, Shenyang, Liaoning, China

**Keywords:** trace elements, cardiovascular disease, Mendelian randomization, mediating effect, multi-omics study, genetic correlation

## Abstract

**Objective:**

Epidemiological evidence indicates that trace elements are significantly associated with cardiovascular health. However, its causality and underlying mechanisms remain unclear. Therefore, this study aimed to investigate the causal relationship between trace elements and cardiovascular disease, as well as their potential mechanism of action.

**Method:**

Two-sample Mendelian randomization (MR) analyses along with mediated and multivariate MR analyses were employed. These analyses utilized 13 trace elements as exposure variables and 20 cardiovascular diseases as outcome variables, with 4907 circulating plasma proteins, 1400 serum metabolites, 731 immune cell phenotypes, and 473 intestinal flora as potential mediators. The Bayesian weighted MR method was used to validate the MR results, and linkage disequilibrium score regression (LDSC) was applied to explore the genetic correlation between trace elements and cardiovascular disease.

**Result:**

Our findings indicated a positive or negative causal relationship between genetically predicted trace elements and cardiovascular disease. An analysis using the Bayesian weighted MR method demonstrated that our causal inference results were reliable. The results of the mediated MR analyses indicate that potassium may reduce the risk of ischemic heart disease by influencing the expression of the plasma proteins BDH2 and C1R. Vitamin B12 may increase the risk of coronary atherosclerosis and cardiovascular death by reducing the levels of VPS29 and PSME1 proteins, while vitamin C may mitigate the risk of cardiac arrest by inhibiting the expression of the TPST2 protein. In addition, potassium can reduce the risk of ischemic heart disease by lowering 4-methoxyphenyl sulfate levels. None of the instrumental variables exhibited pleiotropy in the MR analysis. A sensitivity analysis using the leave-one-out method further confirmed the robustness of our findings. LDSC results indicated a genetic correlation between multiple trace elements and various cardiovascular diseases.

**Conclusion:**

This study uncovered the true causal relationship between trace elements and cardiovascular disease risk using genetic methods, and revealed the significant mediating role of specific plasma proteins and metabolites in this relationship.

## 1 Introduction

Cardiovascular diseases (CVD), which encompass ischemic heart disease, stroke, heart failure, peripheral artery disease, and other cardiac and vascular conditions, have become a leading cause of global morbidity and mortality in the past century (1–3). In 2020, it was estimated that approximately 523 million people worldwide suffered from various CVDs, with 19 million deaths accounting for 32% of total deaths globally, which is an increase of 18.7% compared with 2010 (4, 5). Simultaneously, global disability-adjusted life years and years of life lost due to CVDs have also exhibited a sharp upward trend (6, 7). CVD poses a significant threat to personal health and heavily burdens families and the national economy. In addition to aging and population growth, other factors exacerbate the morbidity and mortality of CVD (8, 9).

In recent years, an increasing number of observational studies have revealed that trace elements play an important role in the prevention and treatment of CVD (10, 11). Although the trace element content in the human body is extremely low, it is an indispensable part of human activity and is of great significance for maintaining the normal physiological function of the heart and stability of the cardiovascular system (12, 13). However, because of unreasonable modern diet structures and environmental pollution, people often struggle to obtain sufficient trace elements from their daily diet, ultimately leading to a deficiency in these key nutrients in the body.

Although several observational studies have consistently shown a significant association between trace elements and CVD, the results of clinical intervention studies remain complex and inconsistent (14, 15). In other words, there may be a potential causal relationship between trace elements and CVD that is yet to be uncovered. Specifically, both insufficient and excessive intake of trace elements can impact the risk of CVDs. To answer these questions, we urgently need to conduct more in-depth research to clarify the causal link between the two and the underlying mechanisms. To enhance our understanding of the significance of trace elements in cardiovascular health, providing theoretical foundations and guidance for the prevention, treatment, and rehabilitation of CVD.

Mendelian randomization (MR) is an effective method for evaluating the causal relationship between observable variable exposure factors, risk factors, and clinical outcomes (16). In particular, this genetic analysis method highlights its importance when randomized controlled trials are limited to verifying causality and observational studies present biased associations owing to potential confounding variables or reverse causality (17). In this study, we systematically collected published data and selected representative trace elements that are widely recognized in previous research. We then adopted the MR analysis method to investigate the genetic associations between trace elements and CVD.

## 2 Data and method

### 2.1 Study design

In this study, we systematically collected data on 13 trace elements and 20 CVDs. We designated the 13 trace elements as exposure factors and the 20 CVDs as outcome variables, and conducted two-sample MR analyses accordingly. Based on the results of the MR analyses, we validated the positive findings using Bayesian weighted Mendelian randomization (BWMR). Subsequently, we performed mediation MR analyses by incorporating four types of omics data—proteomics, metabolomics, immunomics, and microbiomics—as mediator variables to further identify the factors that mediate the causal relationships between the positive exposure factors and the outcomes. Following this, we selected CVDs with multiple positive exposure factors and conducted multivariate Mendelian randomization (MVMR) to assess the risk of each individual factor on the disease outcomes in the context of multiple exposures. Lastly, we comprehensively evaluated the genetic correlations between the 13 trace elements and the 20 CVDs using linkage disequilibrium score regression (LDSC) analysis.

### 2.2 Data source

#### 2.2.1 Source of exposure data

The datasets for selenium and zinc originated from a genome-wide association study (GWAS) encompassing two adult cohorts from Australia and the UK, involving 2603 participants of European descent (18). Data on potassium, calcium, iron, magnesium, retinol, vitamin B6, vitamin B12, folate, vitamin C, vitamin D, and vitamin E were derived from the UK Biobank based on a 24-hour dietary recall questionnaire administered to assess the preceding day’s intake.

#### 2.2.2 Source of outcome data

Datasets pertaining to the 20 CVDs were derived entirely from the 10th version of the FINNGEN database. The FINNGEN database is a specialized bioinformatics platform that amalgamates genotype data from the Finnish Biobank and digital health record data from the Finnish Health Registry. The objective was to collect and assimilate genome-wide association study data specific to the Finnish population, rendering it an exemplary choice for investigating genetic variations associated with disease progression in isolated populations (19).

#### 2.2.3 Source of mediator variable data

Proteomic data were obtained from a large-scale integrated study published by Ferkingstad et al. in 2021 that provided GWAS of plasma protein levels measured with 4,907 aptamers in 35,559 Icelanders (20). Metabolomic data were obtained from a large-scale genome-wide association study of 1,091 blood metabolites and 309 metabolite ratios published by Chen et al. in 2023 (21). The immunomics data were obtained from a GWAS analysis of a dataset of 3,757 European individuals without overlapping samples published by Orrù et al. in 2020, covering 731 exhaustive immunophenotypic classifications. It encompasses 118 absolute cell counts, 389 median fluorescence intensity indicators of surface antigen levels, 32 morphological parameters, and 192 relative cell counts (22). Microbiome data came from a single cohort GWAS study of 5,959 European individuals published by Qin et al. in 2022, which used strict criteria to remove low-quality samples and variants, and determined the genome-wide association between human genotype and gut microbial abundance (23).

All participants provided informed consent for the corresponding original studies. These large-scale and rich datasets laid the foundation for subsequent genetic information analyses.

### 2.3 Study method

#### 2.3.1 MR analysis method

The role of Single Nucleotide Polymorphisms (SNPs) as instrumental variables is crucial in the study of causal effects based on genetic variation because they are directly related to the accuracy of causal inference. Therefore, the selected SNPs must strictly adhere to three core assumptions: First, they must be closely related to the studied exposure factors; second, their association with outcomes should not be influenced by confounding factors; and finally, these SNPs can only be linked to clinical outcomes through exposure factors (24). To exclude the potential linkage disequilibrium bias, we specified strict criteria for screening SNPs that were significantly associated with exposure factors. Specifically, we required all SNPs with data to have R² values lower than 0.001 in the linkage disequilibrium analysis and genetic distances greater than 10,000 kb. Such screening criteria ensure the independence of the selected SNPs, thereby enhancing the robustness of subsequent causal inference (25). We also used the F-statistic to evaluate the strength of the selected instrumental variables (26). When the F-statistic exceeds 10, there is no weak instrumental variable bias, ensuring the validity of the causal inference (27). We also excluded SNPs with mismatched alleles, palindromic SNPs, and those with missing values. We also set a stringent statistical significance threshold (p < 5e-06).

Upon obtaining the genetic dataset pertaining to the outcome variables, we extracted SNPs that exhibited significant associations with the exposure factors and treated them as outcome instrumental variables. We then thoroughly recorded crucial information for each selected SNP, including the effect allele (EA), allele effect value (β), standard error (SE), and P value.

The R statistical software (version 4.3.3) and associated R packages were used to perform comprehensive statistical analysis of the data and construct the genetic relationship network. During the analysis, we employed a range of MR statistical methods, including the inverse variance weighted (IVW), weighted median, MR-Egger, and simple and weighted mode methods. In selecting the analysis methods, the IVW method was considered the core approach for MR analysis. This method aggregates the MR effect estimates of individual SNPs to obtain a comprehensive weighted result for causal effect (28). When the IVs exhibit no pleiotropy, the analysis results derived using the IVW method are highly reliable (29). Furthermore, in actual research, instrumental variables may fail, and the weighted mode method can enhance the robustness of a study. This method can accurately estimate causal effects, even when up to 50% of the information originates from invalid instrumental variables (30).

To clarify whether pleiotropy was present in the instrumental variables, we performed an MR-Egger regression analysis. This method intuitively displays the effect estimates of pleiotropy through an intercept, providing an effective means of evaluation (31). Pleiotropy indicated that, in addition to the expected exposure factors, SNPs may also directly affect the results through other pathways or mechanisms. Moreover, pleiotropy affects the accuracy of MR analysis because it violates the core hypothesis of MR analysis, that is, genetic variation can only affect the results through exposure factors. Simultaneously, the MR-Presso test was employed to further detect and correct pleiotropy by removing abnormal SNPs (32). Cochran’s Q test was used to evaluate and quantify the heterogeneity of the IVs. Heterogeneity may result from differences in the experimental design and methodology, population stratification, genetic background, or the interaction of complex factors. In cases where heterogeneity existed, particularly when the p-value was < 0.05, the IVW random effects model was selected to estimate the causal effect, ensuring the robustness of the results. To evaluate the stability of our results and prove that the causal effect does not depend on a single instrumental variable, we used the leave-one-out method for sensitivity analysis. The core of this method is to eliminate each SNP individually and then recalculate the joint effect of the remaining variables for comparison with baseline results. Thus, we observed changes in the results after removing each variable (33).

#### 2.3.2 BWMR analysis method

We used the BWMR analysis to verify the results of the two-sample MR. BWMR is a statistical method that integrates Bayesian inference and MR. MR was employed to eliminate the influence of confounding factors, whereas Bayesian inference combined prior information with observational data to obtain a posterior distribution, thereby estimating causality more accurately (34). This method assigns weights to the effect sizes of the exposure factors based on prior information and sample data, ensuring that exposure factors with larger effects carry greater weights in the analysis.

#### 2.3.3 Mediation MR analysis method

The mediation MR analysis method is a two-step approach that identifies the factors mediating the relationship between exposure factors and outcomes, suggesting that intervention in these mediators may reduce the impact of exposure factors on outcomes (35). The causal effect of exposure factors on outcomes can be divided into two parts: one is the direct impact of exposure factors on outcomes; the other part is the mediation effect, meaning that the influence of exposure factors on outcomes operates solely through this mediation effect (36). To estimate the effect size of each mediator, we employed the product of coefficients method (37). This approach initially calculates the effect size (beta1) of the trace element on the mediator, and subsequently determines the effect size (beta2) of the mediator on the outcome. The product of beta1 and beta2 is taken as the indirect effect, representing the impact of trace element on the corresponding cardiovascular disease through the mediator (38). we set a strict statistical significance threshold (p < 1e-05, with the proteomic significance threshold set at p < 5e-08).

The false discovery rate (FDR) is a measure used to identify decision-making errors in hypothesis testing. Specifically, FDR represents the proportion of all rejected null hypotheses (i.e. those considered significant findings) that are false (i.e. the original null hypothesis is true) (39). It serves as a crucial indicator in the large-scale data analysis of various omics. By controlling the FDR, it becomes feasible to accurately identify truly meaningful findings and avoid erroneous conclusions resulting from accidental factors (40). In this study, to control the proportion of false positives and enhance the confidence in the results of multi-omics data analysis, we set the FDR filtering threshold to less than 0.1.

#### 2.3.4 MVMR analysis method

MVMR, based on the principles of MR, represents a specialized extension of the MR methodology (41). When multiple variables need to be considered, MVMR offers an effective analytical approach. By simultaneously evaluating the direct risks of each factor on disease outcomes under the influence of multiple exposures, MVMR provides a more comprehensive analysis of the complex associations between variables, thereby enhancing our understanding of the mechanisms underlying disease onset and progression (42, 43).

#### 2.3.5 LDSC analysis method

LDSC is a statistical method used to estimate the genetic contributions to complex diseases and traits without bias due to sample overlap (44). The inflated contributions from true polygenic signals or biases were quantified by examining the association between the statistics and linkage disequilibrium, which was calculated based on the genetic correlation and physical distance between SNP pairs (45). We filtered the GWAS summary measures according to hapmap3 and excluded SNPs if their chains were ambiguous or the allele frequency was <0.01. Subsequently, we used precalculated linkage disequilibrium scores and weights for European populations based on the 1000 genomes project (46). The Z-score of each variant in the exposure group was multiplied by the Z-score of each variant in the outcome group. Genetic covariance was estimated by regressing the product on the LD score. Genetic covariance normalized by SNP heritability represents genetic correlation (47).

## 3 Results

### 3.1 MR analysis result

**Figure 1:**
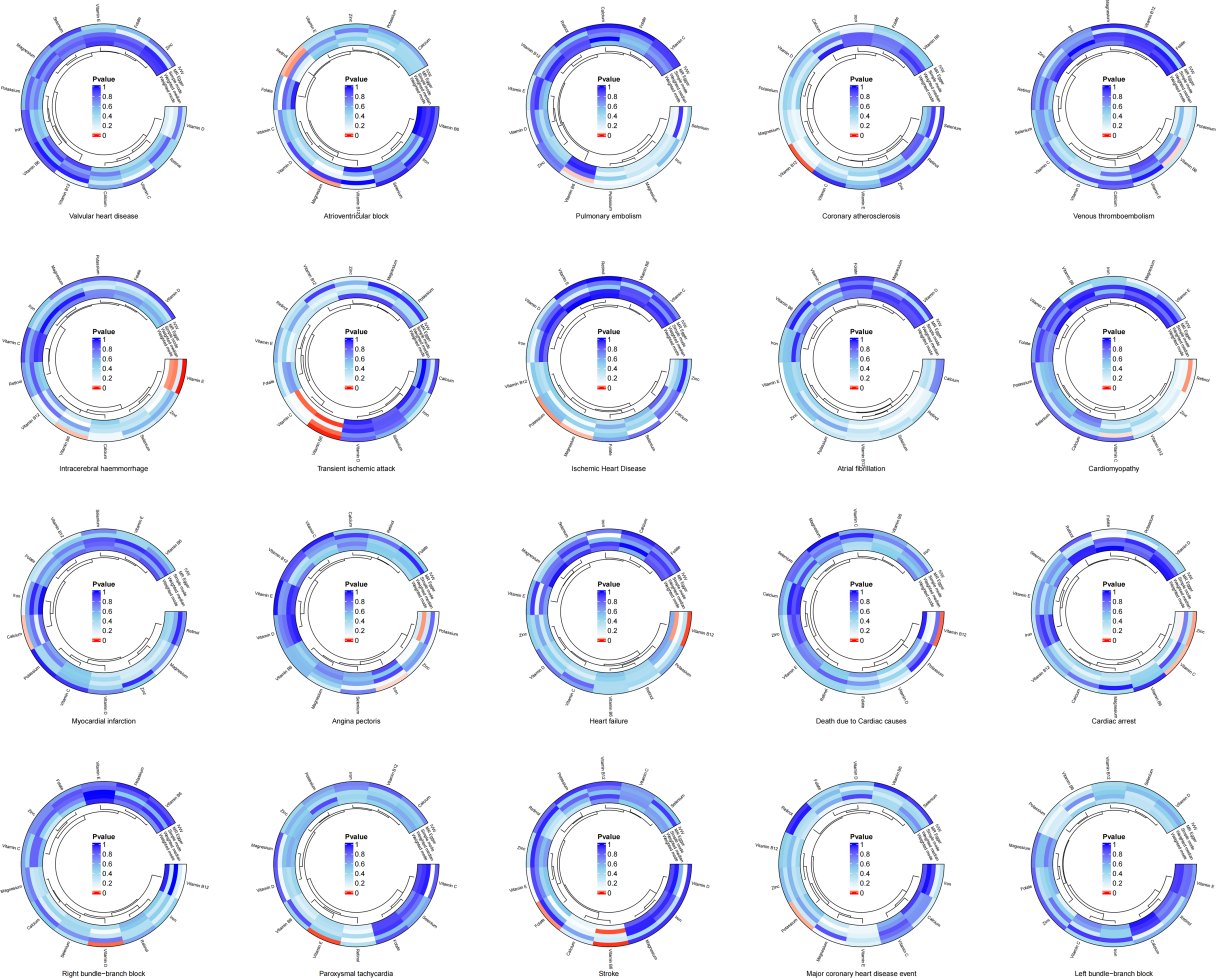
Circular diagram of genetic link between trace elements and cardiovascular diseases.

The results of the two-sample MR analysis revealed a potential genetic link between trace elements and CVD risk. To illustrate this relationship more intuitively, we constructed a circular diagram depicting trace elements and CVD (**Figure 1**). Specifically, preliminary MR results indicated significant negative correlations between magnesium and atrioventricular block (beta = -0.455, OR = 0.634, 95% CI: 0.427–0.942, p = 0.024) and ischaemic heart disease (beta = -0.141, OR = 0.868, 95% CI: 0.763–0.988, p = 0.032). Potassium decreases the risk of ischaemic heart disease (Beta = -0.177, OR = 0.838, 95% CI: 0.719–0.976, p = 0.023) and major coronary heart disease events (Beta = -0.218, OR = 0.804, 95% CI: 0.662–0.978, p = 0.029). Calcium levels exhibits a significant positive relationship with myocardial infarction (beta = 0.215, OR = 1.239, 95% CI: 1.016–1.512, P = 0.034). Iron level was significantly and negatively associated with angina pectoris (beta = -0.208, OR = 0.812, 95% CI: 0.666–0.989, p = 0.039). Excessive intake of zinc increases the risk of cardiac arrest (Beta = 0.158, OR = 1.171, 95% CI: 1.019–1.345, p = 0.026). Retinol displays a notable positive relationship with atrioventricular block (Beta = 0.495, OR = 1.640, 95% CI: 1.042–2.583, p = 0.033). Vitamin B6 demonstrates negative causal relationships with transient ischaemic attack (Beta = -0.337, OR = 0.714, 95% CI: 0.577–0.883, p = 0.002), intracerebral haemmorrhage (Beta = -0.506, OR = 0.603, 95% CI: 0.379–0.960, p = 0.033), and stroke (Beta = -0.280, OR = 0.755, 95% CI: 0.625–0.913, p = 0.004). Vitamin B12 is associated with an increased risk of coronary atherosclerosis (Beta = 0.237, OR = 1.268, 95% CI: 1.059–1.518, p = 0.010), heart failure (Beta = 0.297, OR = 1.346, 95% CI: 1.086–1.668, p = 0.007), and death due to cardiac causes (Beta = 0.376, OR = 1.456, 95% CI: 1.084–1.956, p = 0.013). In contrast, folate reduced the risk of stroke (beta = -0.219, OR = 0.803, 95% CI: 0.667–0.968, P = 0.021). Vitamin C exhibited a negative causal relationship with cardiac arrest (beta = -0.789, OR = 0.454, 95% CI: 0.220–0.936, P = 0.032). Vitamin D showed a positive causal relationship with right bundle branch block (beta = 1.134, OR = 3.109, 95% CI: 1.271–7.605, P = 0.013). Lastly, vitamin E demonstrates negative causal relationships with intracerebral haemmorrhage (Beta = -0.714, OR = 0.490, 95% CI: 0.327–0.733, p = 0.001) and paroxysmal tachycardia (Beta = -0.382, OR = 0.683, 95% CI: 0.516–0.902, p = 0.007).

**Figure 2:**
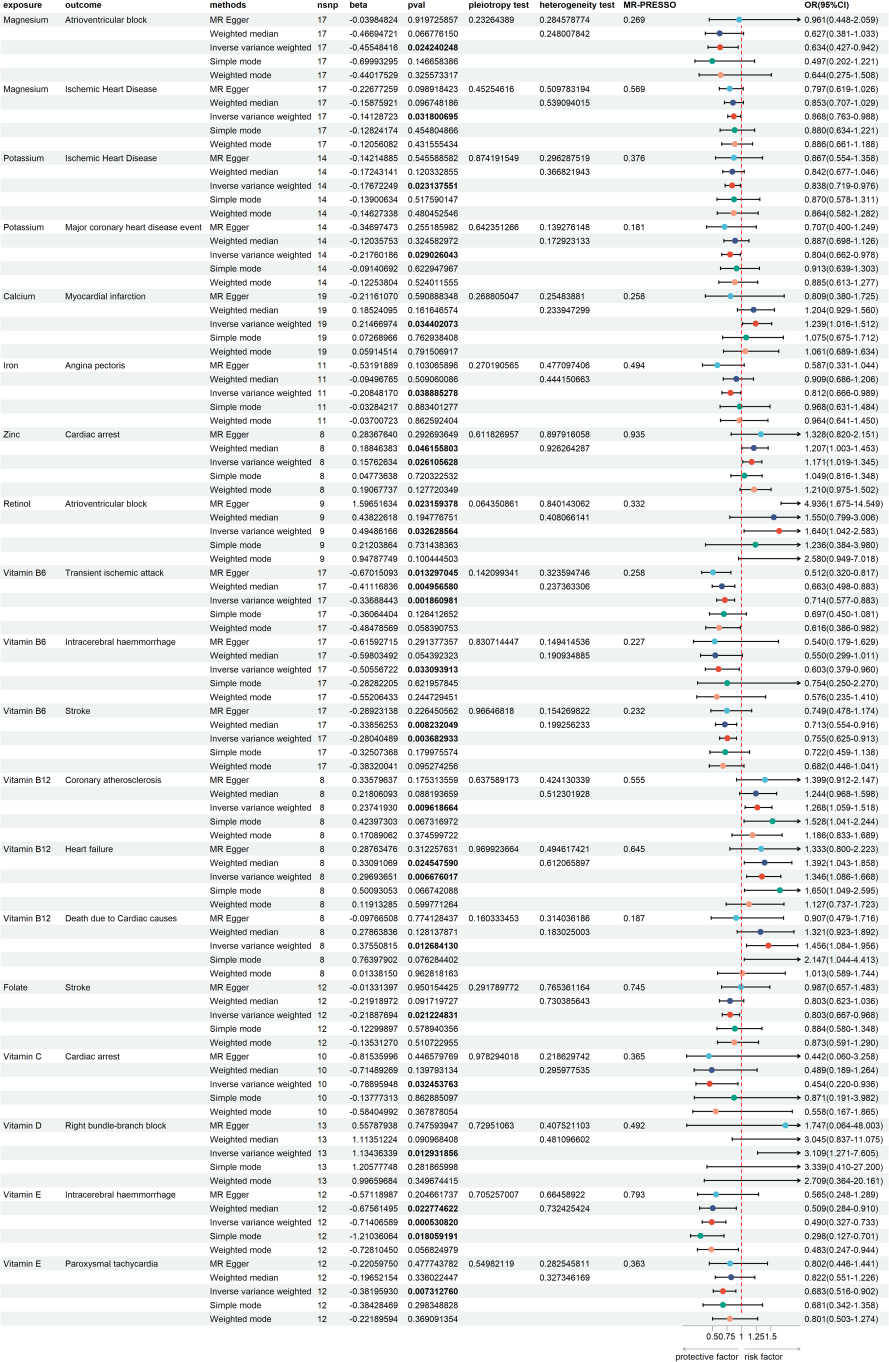
Forest plot of trace elements with significant causal relationships to cardiovascular diseases.

The test results indicated that the SNPs of the instrumental variables did not exhibit horizontal pleiotropy. Additionally, we employed IVW and MR-Egger regression methods to detect heterogeneity among the instrumental variables. Using the leave-one-out method for sensitivity analysis, we found that the results were highly consistent with the MR results, indicating that our conclusion was based on the joint action of multiple instrumental variables rather than relying on a single factor. The results of the MR analysis are presented in **Supplementary Table 1**. To visually present the significant causal relationships and the results of the various sensitivity analyses, we constructed a forest plot illustrating trace elements with significant causal relationships to CVDs (**Figure 2**).

### 3.2 BWMR analysis result

Subsequently, we employed the BWMR method to verify the results of the previous analysis. The validation results obtained using this method indicate that the causality conclusion derived from our earlier MR analysis was significant and reliable (**Supplementary Table 2**). We further explored the potential mechanisms underlying these causal relationships.

### 3.3 Mediation MR analysis result

To investigate the potential mediators in the established causal relationships between the aforementioned trace elements and cardiovascular diseases, we employed a two-step mediation analysis approach. We first treated the SNPs derived from the four types of omics data as the exposures and cardiovascular disease as the outcome. To further enhance the reliability of the analysis results, we required consistency in the directionality across all five MR methods, with the IVW method being selected as the primary approach to estimate causal effects. With the FDR threshold set at less than 0.1, we found that plasma proteins were associated with 11 CVDs, serum metabolites with 6 CVDs, immune cell phenotypes with 3 CVDs, and intestinal flora with only one CVD. The sensitivity analysis results did not indicate that the SNPs of the instrumental variables showed pleiotropy. The results of FDR filtering are shown in **Supplementary Table 3**.

**Figure 3:**
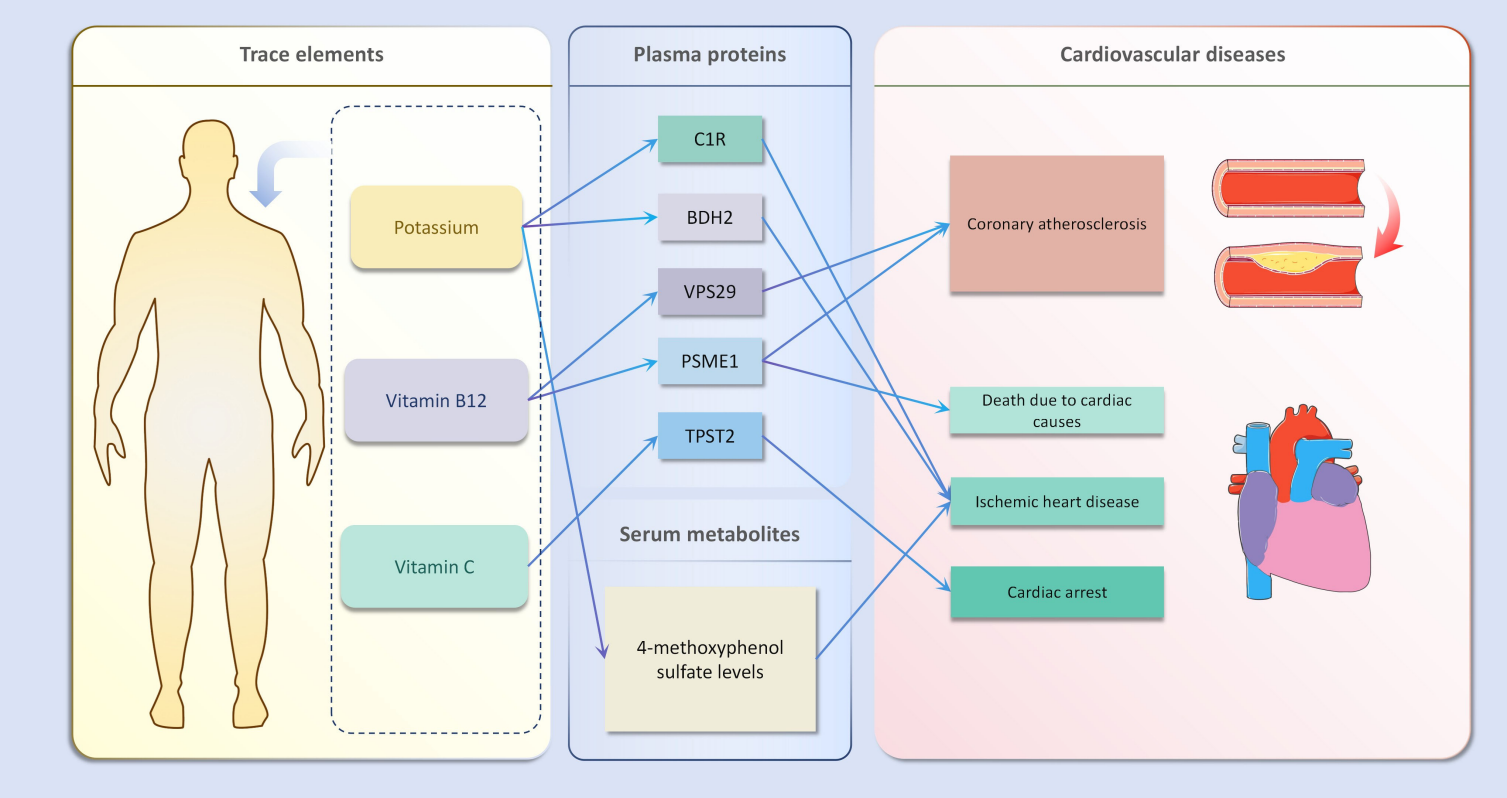
Schematic diagram of mediation mr results.

**Figure 4:**
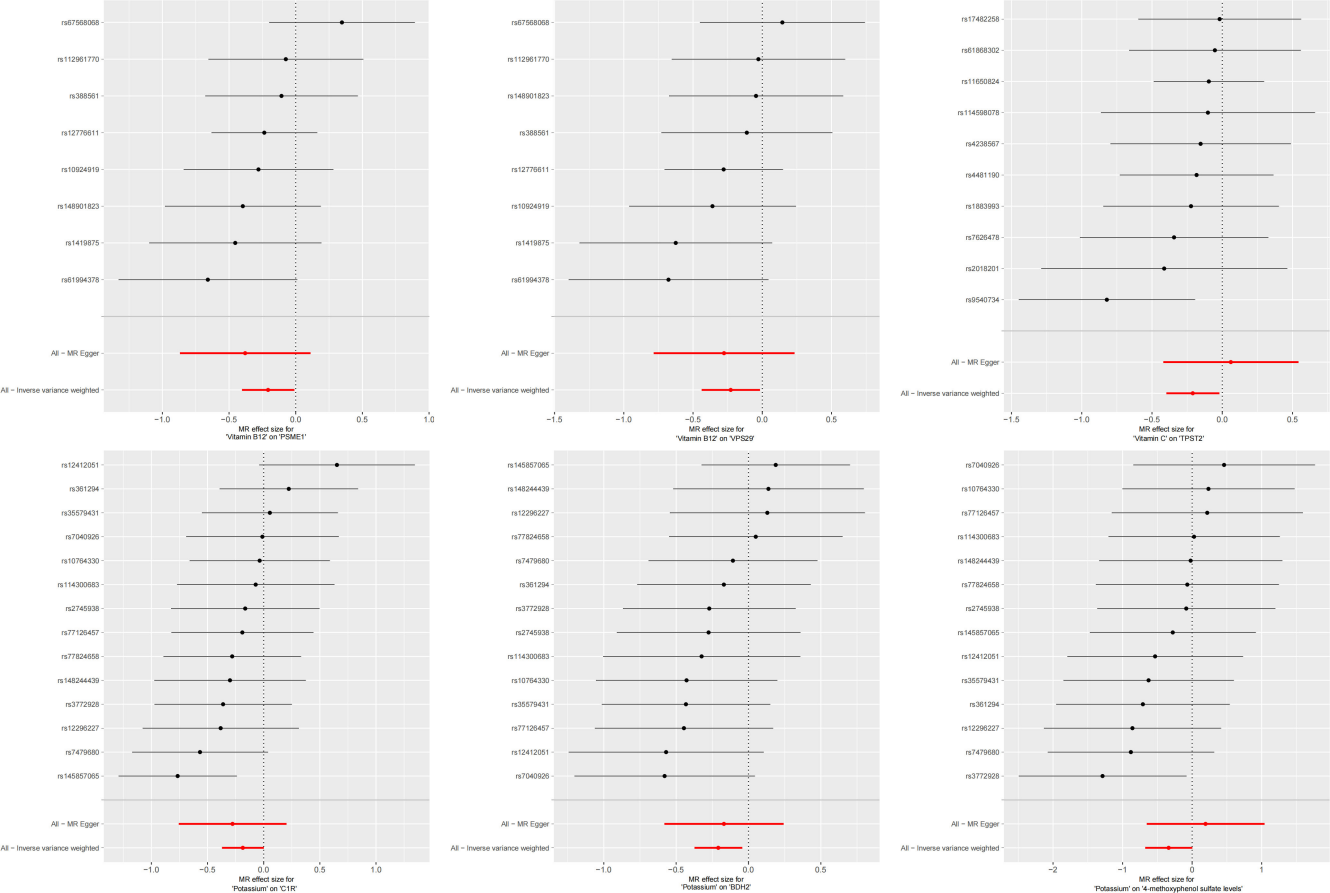
Summary forest plot of mediation mr results.

**Figure 5:**
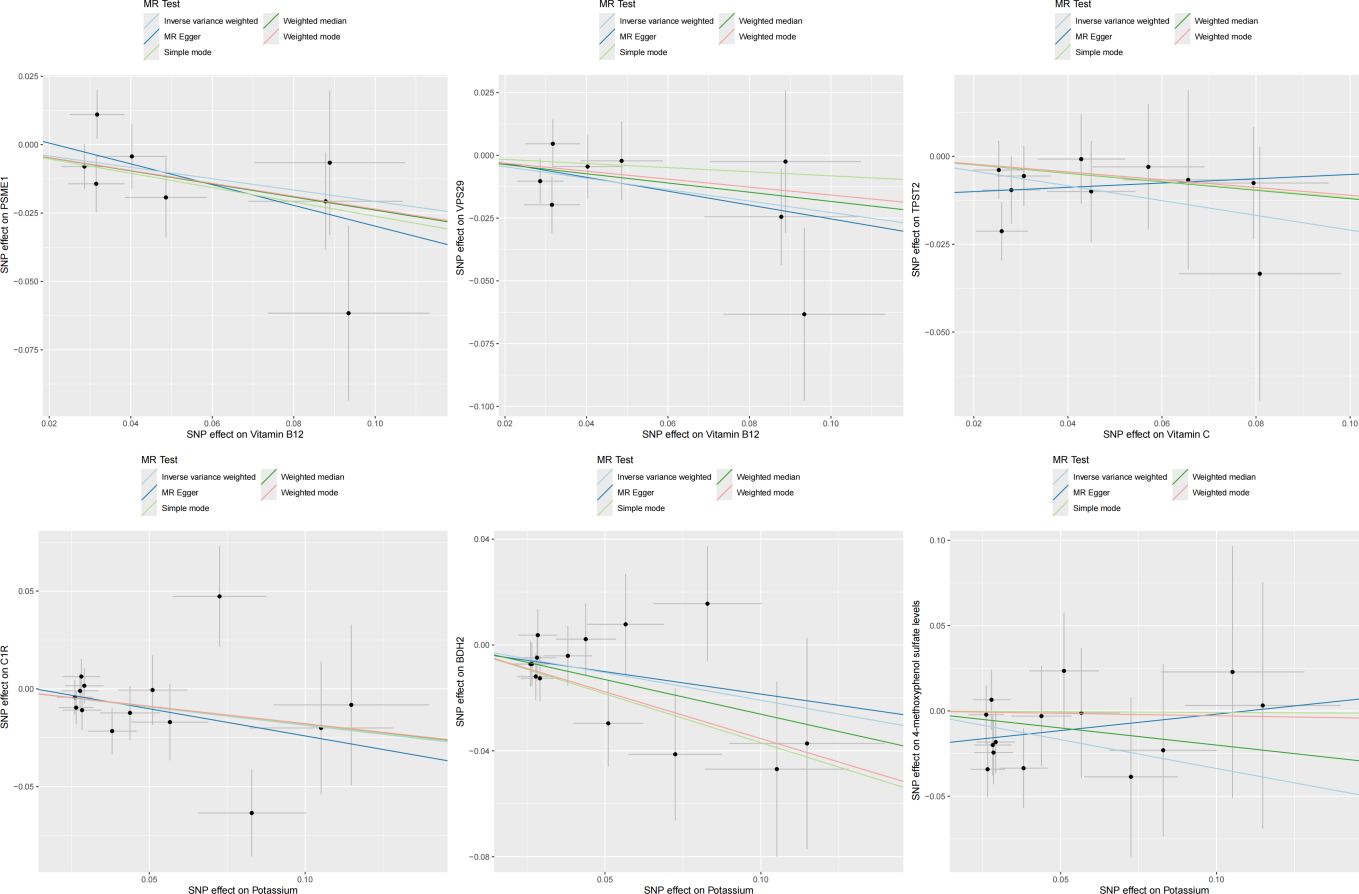
Summary scatter plot of mediation mr results.

**Figure 6:**
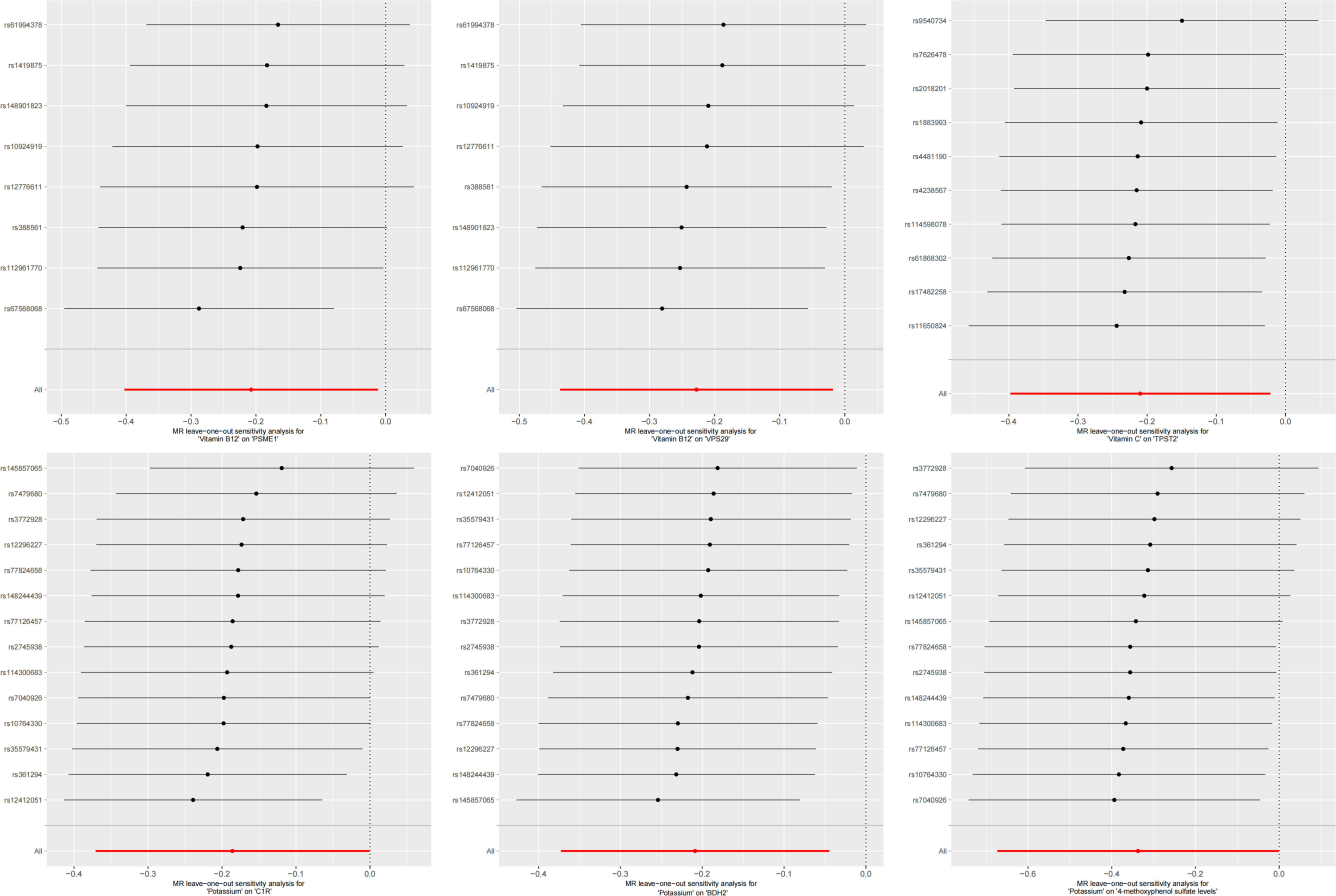
Summary leave-one-out sensitivity analysis plot of mediation mr results.

In the second step of the analysis, we will designate the multi-omics data that exhibited significant genetic causality with CVD in the first step as the outcome variables, and treat the trace elements that have a causal relationship with the corresponding cardiovascular disease as the exposure variables. Integrating the results from the two-step mediation analysis, we have successfully constructed two causal chains: “trace elements → plasma proteins → CVD” and “trace elements → serum metabolites → CVD”. In detail, potassium reduced the risk of ischaemic heart disease by decreasing the expression of C1R (Mediated Effect=-0.014, 95% CI: -0.014–-0.013; Mediated Proportion=7.82%, 95% CI: 7.48%-8.17%) and BDH2 (Mediated Effect=-0.025, 95% CI: -0.025–-0.024; Mediated Proportion=13.89%, 95% CI: 13.40%-14.37%). Vitamin B12 may increase the risk of coronary atherosclerosis by inhibiting VPS29 (Mediated Effect=0.031, 95% CI: 0.030–0.032; Mediated Proportion=13.04%, 95% CI: 12.53%-13.56%) and PSME1 (Mediated Effect=0.101, 95% CI: 0.097–0.106; Mediated Proportion=42.75%, 95% CI: 40.88%-44.61%). Moreover, the inhibition of PSME1 expression by vitamin B12 may also elevate the risk of death caused by heart disease (Mediated Effect=0.054, 95% CI: 0.053–0.055; Mediated Proportion=14.39%, 95% CI: 14.05%-14.74%). Vitamin C may reduce the risk of cardiac arrest by inhibiting the expression of the TPST2 (Mediated Effect=-0.111, 95% CI:7nbsp;-0.119–-0.103; Mediated Proportion=14.09%, 95% CI: 13.09%-15.09%). Potassium can also reduce the risk of ischemic heart disease by reducing 4-methoxyphenyl sulfate levels (Mediated Effect=-0.022, 95% CI: -0.029–-0.016; Mediated Proportion=12.66%, 95% CI: 8.94%-16.38%). We did not find evidence of immunomics- or microbiomics-mediated effects of trace elements on cardiovascular disease. The results of the second step of the analysis, along with the mediation effect results, are presented in **Supplementary Table 4**. The schematic diagram of “trace elements → plasma proteins/serum metabolites → CVDs” is shown in **Figure 3**. A forest plot summarizing the MR results is shown in **Figure 4**. A scatter plot summarizing the MR results is shown in **Figure 5**. A leave-one-out sensitivity analysis plot summarizing the MR results is shown in **Figure 6**.

### 3.4 MVMR analysis result

We conducted a MVMR analysis on CVD with multiple positive exposure factors. The results of the MVMR analysis indicated that an increase in retinol intake significantly elevated the risk of atrioventricular block (beta = 0.558; OR = 1.747; 95% CI, 1.086–2.811; p = 0.021). Conversely, an increase in magnesium intake markedly reduced this risk (beta = -0.621, OR = 0.537, 95% CI: 0.380–0.759, p = 0.0004). The effects of potassium (p = 0.281) and magnesium (p = 0.566) on ischemic heart disease became nonsignificant. Similarly, no significant correlations were observed between folate (p = 0.606) and vitamin B6 (p = 0.120) levels and stroke. Furthermore, an increase in zinc intake was found to increase the risk of cardiac arrest (beta = 0.173, OR = 1.189, 95% CI: 1.067–1.324, p = 0.002), while vitamin C intake reduced this risk (beta=-0.919, OR=0.399, 95% CI: 0.200–0.795, p = 0.009). Vitamin E intake significantly decreased the risk of intracerebral haemmorrhage (beta=-0.666, OR=0.514, 95% CI: 0.312–0.845, p = 0.009), whereas no significant causal relationship was observed between vitamin B6 and the reduction of intracerebral haemmorrhage risk (p = 0.666). There was no evidence of pleiotropy or heterogeneity in the MVMR results. The results of the MVMR analysis are shown in **Supplementary Table 5**.

### 3.5 LDSC analysis result

**Figure 7:**
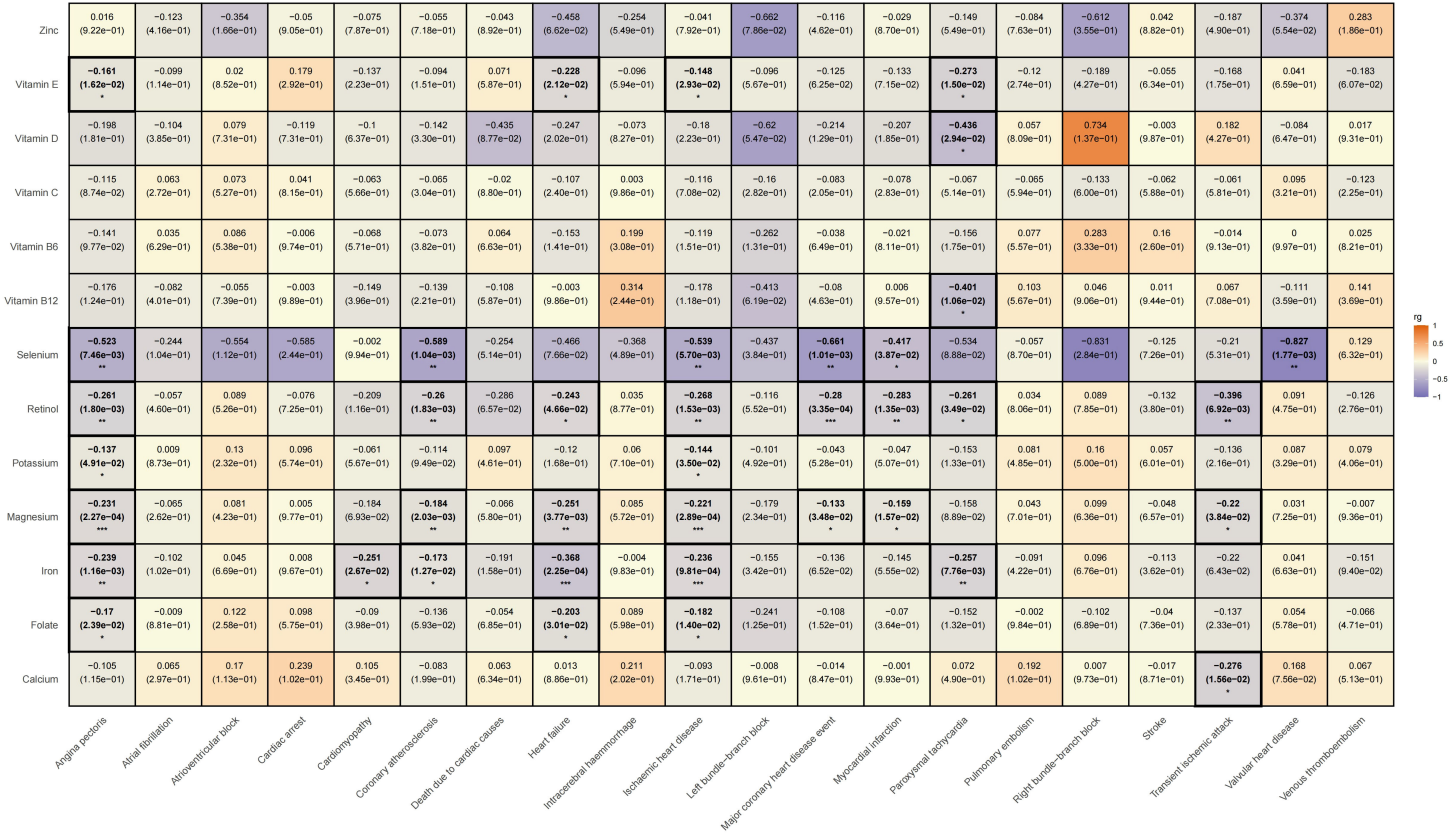
Heatmap of ldsc results for the effects of trace elements on cardiovascular diseases.

We subsequently conducted an LDSC analysis, which revealed a genetic correlation between various trace elements and CVD. Compared to previous two-sample MR results, the LDSC findings further indicate that there is a significant negative genetic correlation between iron and angina pectoris (rg = -0.239, rg_p = 0.001); potassium (rg = -0.144, rg_p = 0.035) and magnesium (rg = -0.221, rg_p < 0.001) show negative genetic correlations with ischemic heart disease; and vitamin E exhibits a negative genetic correlation with paroxysmal tachycardia (rg = -0.273, rg_p = 0.015). However, it must be acknowledged that we did not find evidence of genetic correlation between trace elements and specific cardiovascular conditions, such as atrioventricular block, pulmonary embolism, intracerebral haemmorrhage, deep vein thrombosis, atrial fibrillation, cardiovascular death, cardiac arrest, stroke, left bundle branch block, and right bundle branch block. Please refer **Supplementary Table 6** for specific LDSC results. The LDSC heatmap is shown in **Figure 7**.

## 4 Discussion

In this study, we successfully identified 19 causal relationships between 13 trace elements and 20 CVDs using the MR method and a large-scale published genetic dataset. As a unique genetic tool, the MR method can provide insights into genetic variations closely related to risk factors (48). Through Bayesian weighted MR validation, we further confirmed the robustness of the study results. To explore the mechanisms underlying these associations, we performed a mediated MR analysis using four omics genetic datasets. We found that potassium decreased the risk of ischaemic heart disease by reducing C1R and BDH2 expressions. Vitamin B12 may increase the risk of coronary atherosclerosis by inhibiting the expression of VPS29 and PSME1 proteins, and elevate the risk of death caused by heart disease through the suppression of PSME1 expression. Vitamin C may reduce the risk of cardiac arrest by inhibiting TPST2 expression. Our study also showed that potassium reduced the risk of ischaemic heart disease by reducing the levels of 4-methoxyphenol sulfate.

### 4.1 Mineral

Potassium plays a pivotal role in maintaining the functions of numerous human cells (49). It achieves a negative sodium balance by promoting sodium excretion and subsequently regulating the electrolyte balance in body fluids (50). Potassium alleviates oxidative stress, augments nitric oxide synthesis, safeguards endothelial cells and reduces vascular stiffness (51). It halts the assembly of the NLRP3 inflammasome and suppresses the release of the pro-inflammatory cytokine interleukin-1β, thereby mitigating the inflammatory response (52). Furthermore, it reduced the adhesion of macrophages to the vascular wall and alleviated atherosclerotic plaque (53). Potassium transmits nerve impulses and controls myocardial contractions by preserving myocardial membrane potential for electrical excitation (54). Several prospective studies and extensive meta-analyses have consistently demonstrated that increasing dietary potassium intake has a profound positive effect on CVD risk factors and overall cardiovascular health (55, 56).

β-hydroxybutyrate dehydrogenase 2 (BDH2) is an enzyme that plays a pivotal role in citric acid cycle metabolism and ketogenesis. It belongs to the short-chain dehydrogenase family and plays a significant role in various biological and pathological processes, particularly in the utilization of cytosolic ketone bodies, regulation of immune cells, and tumour progression (57, 58). Recent studies have revealed an important role of BDH2 in apoptosis and autophagy. Specifically, BDH2 can regulate the level of reactive oxygen species in cells via the keap1/nrf2/are signaling pathway, further influencing the pi3k/akt/mtor signaling pathway and significantly inducing apoptosis and autophagy in gastric cancer cells (59). In hepatoma cells, BDH2 can promote mitochondrial apoptosis by upregulating the pro-apoptotic protein Bax and downregulating the anti-apoptotic protein Bcl-2 and can also induce apoptosis of liver cancer cells through a caspase 3-independent pathway (60). These findings provide clues about the possible mechanism of BDH2 in CVD: elevated expression of BDH2 increases the risk of ischaemic heart disease, which may be mediated through the aforementioned mechanisms of apoptosis and autophagy.

Complement component 1R (C1R), a modular serine protease composed of enzymes, plays a pivotal role in the classical complement system (61). As an auto-activating component of the C1 complex, it can combine with C1s and C1q to form the C1 complex and serve as the initiator of the classical pathway of the immune system (62). The complement system, which consists of approximately 35 soluble cell surface proteins, is a key component of the innate immune system (63). It recognizes and eliminates invading microorganisms or abnormal host cells through interactions, and plays a crucial role in triggering inflammatory responses and regulating adaptive immunity (61, 64). The complement system can influence a range of biological processes, including extracellular matrix remodeling, endoplasmic reticulum stress, inflammatory response, oxidative stress, vascular calcification, and apoptosis, through various mechanisms, such as chemotaxis, phagocytosis, anaphylaxis, cytolysis, and autophagy (65–67). These processes may lead to thrombosis, endothelial dysfunction, vascular remodeling, atherosclerosis, hyperglycemia, and hypertension, ultimately increasing the risk of CVD (68).

4-methoxyphenol sulfate is the sulfate form of 4-methoxyphenol and is also known as 4-hydroxyanisole or p-methoxyphenol. Previous studies have revealed that 4-methoxyphenol can inhibit the growth of tumour cells, demonstrating significant cytotoxicity (69). In a previous study, p-methoxyphenol was directly injected into the substantia nigra of the rat brain. The results indicated that the function and structure of dopamine neurones were damaged, and the extent of damage correlated with the dose (70). This suggests the potential toxicity of 4-methoxyphenol to the nervous system *in vivo* and implies that, in the blood circulation, the substance may exert direct or indirect effects on the cardiovascular system. It is worth noting that neurotransmitters such as dopamine can indirectly regulate cardiovascular function by influencing the autonomic nervous system, thereby affecting vasomotor activity and heart rate (71).

Magnesium is the fourth most abundant mineral and the second most abundant cation in the human body (72). As an important cofactor in more than 600 enzymatic reactions, Mg participates in numerous physiological processes including DNA and RNA synthesis, energy metabolism, protein synthesis, fatty acid metabolism, and nearly all hormonal reactions (73). In the heart, magnesium regulates neuronal excitation, controls myocardial contraction, inhibits smooth muscle cell proliferation and migration, and reduces atherosclerosis and thrombosis by regulating various ion transporters (74). Epidemiological studies, randomized controlled trials, and meta-analyses have found a negative correlation between magnesium intake and the risk of CVDs (75).

Iron plays an indispensable role in several biological processes, serves as a cofactor for several enzymes and is a crucial component of hemoglobin synthesis in red blood cells. It plays a pivotal role in oxygen transport and cell respiration, and participates in redox reactions, cell growth, maintenance, and repair (76). It also influences skeletal muscle metabolism, immune and nervous system functions, lipid metabolism, and other biological processes (77). Studies have demonstrated that iron deficiency is associated with an increased risk of heart failure and coronary atherosclerotic heart disease (78). Iron deficiency can result in ventricular dilatation, mitochondrial ultrastructural distortion, and release of cardiac cytochrome c (79). Therefore, maintaining an appropriate dietary iron intake is important for preventing CVDs.

Calcium is the most abundant mineral element in the human body and plays a pivotal role in numerous physiological processes, including cell function, nerve signal transmission, blood coagulation, vascular activity regulation, exocytosis, and hormone secretion (80, 81). Although calcium is essential for the human body, excessive calcium intake may pose health risks (82). Excessive calcium intake may lead to cardiovascular calcification, resulting in hardening and narrowing of the vascular wall and may also induce vascular inflammation and thrombosis, thereby increasing the risk of CVD and mortality (83). Furthermore, excessive calcium intake may also induce transient hypercalcemia, triggering a cascade of calcium-mediated coagulation reactions (83) that subsequently lead to increased blood pressure and alterations in endothelial function (84, 85). Ultimately, this could exacerbate arteriosclerosis and increase the burden on the heart. Several large-scale prospective studies and randomized controlled trials have demonstrated that an increased intake of calcium supplements may be associated with an increased risk of mortality from CVD (86–88).

Zinc is an essential trace element that binds to various enzymes and transcription factors, influencing processes such as transcription, RNA and DNA synthesis and repair, protein synthesis, cell cycle progression, apoptosis, as well as glucose and lipid metabolism, and immune regulation (89, 90). In the cardiovascular system, zinc promotes vasodilation by modulating transient receptor potential channels, facilitating prostaglandin production, and inhibiting calcium channels in smooth muscle (91). Additionally, zinc reduces oxidative stress in the cardiovascular system by minimizing free radical damage, preventing lipid peroxidation, and mitigating myocardial ischemia-reperfusion injury (92). Zinc can also enhance endothelial function by regulating nitric oxide synthase activity (93). However, the association between zinc intake and cardiovascular risk has been inconsistent in previous research. Some studies have indicated no significant relationship between zinc intake and CVD (94). Several studies have even demonstrated a positive correlation between dietary zinc intake and the incidence of CVD, CVD mortality, and all-cause mortality (95–97).

### 4.2 Vitamin

Retinol, also known as vitamin A1, is a fat-soluble vitamin; however, its comprehensive biological activity remains poorly understood (98). Numerous large intervention trials investigating the relationship between retinol and CVDs have reported inconsistent findings (99, 100). Our study revealed that retinol use is associated with an increased risk of atrioventricular block. This may be because retinol plays a pro-oxidant role under specific conditions, thus triggering an inflammatory response in the myocardium, further promoting the progression of myocardial hypertrophy and fibrosis, and upregulating the expression of receptors for advanced glycation end products (101, 102). Consequently, the disorder of intercellular junctions in the myocardium leads to changes in myocardial tissue structure and affects cell membrane potential and ion channel function, which results in changes in electrical conduction characteristics and ultimately induces the occurrence of atrioventricular block (103).

Vitamin B6 is a widely utilized coenzyme that participates in a variety of biochemical reactions, it is deeply involved in gene expression, amino acid conversion, neurotransmitter production, synthesis of DNA and RNA basic components, maintenance of fluid balance, normal functioning of the immune system, and also plays a key role in glucose and lipid metabolism (104). Several studies have shown that vitamin B6 exerts significant cardiovascular protective effects, which can be attributed to its antioxidant and anti-inflammatory properties (105). Specifically, it actively participates in the synthesis of nitric oxide by reducing homocysteine levels and effectively regulating blood lipid levels, particularly triglycerides and low-density lipoprotein cholesterol (106–108). This not only aids in improving blood circulation but also reduces the risk of thrombosis.

Folate, also known as vitamin B9 or pteroylglutamic acid, is a water-soluble B vitamin that plays multiple key roles in organisms (109). It participates in the synthesis of nucleic acids, regeneration of methionine, and transfer and utilization of one-carbon units (110). Additionally, it plays a crucial role in biological processes such as the synthesis and methylation of DNA and RNA, generation of antioxidants, and epigenetic regulation (111). Insufficient folate intake leads to increased Hcy levels, which are closely associated with endothelial dysfunction and atherosclerosis (112). While there is a viewpoint that reducing homocysteine levels has no practical benefit for cardiovascular health, it is worth noting that folate deficiency can trigger methylation disorders, particularly the hypomethylation of DNA and other molecules, potentially contributing to the development of CVD (113). Notably, folate has also been found to reverse endothelial dysfunction, an effect not achieved solely by reducing homocysteine levels (114). Furthermore, folate alleviates oxidative damage by directly scavenging superoxide free radicals, thereby exerting protective effects on endothelial function (115, 116).

Vitamin B12 (cobalamin), the only vitamin containing metal elements, serves as a cofactor for methionine synthase and methylmalonyl-CoA in mammals (117). Although numerous studies have associated low vitamin B12 levels with an increased risk of cardiovascular and cerebrovascular diseases, attributing this link to hyperhomocysteinemia or an inflammatory response, this viewpoint is controversial (118, 119). Recent studies have presented contrary evidence with several studies demonstrating that there is no significant association between vitamin B12 intake and CVD incidence, mortality, or all-cause mortality. Some studies have even indicated that excessive intake of vitamin B12 may increase these risks (120–122).

Membrane vesicle protein sorting-related protein 29 (VPS29) is the core component of retromer complex (123). Together with VPS26 and VPS35, it forms a cargo-recognition trimer, a highly conserved eukaryotic protein complex (124). The retromer plays a key role in eukaryotes by managing the retrieval pathway from endosomes to the trans-Golgi network, localizing to endosomes, and is responsible for the precise sorting of transmembrane protein cargo into vesicles and elongated tubular structures (125, 126). Furthermore, it drives recycling of metazoan cell plasma membranes (125). Although no direct study has explored the association between VPS29 and CVD, based on existing evidence, we can reasonably speculate that VPS29 may play a positive role in inhibiting CVD. This mechanism may be closely related to its key role in maintaining normal lysosomal function, regulating cellular homeostasis, and protecting neuronal health (127, 128).

Proteasome activator subunit 1 (PSME1) is a protein composed of 248 amino acids with a molecular weight of approximately 29kDa (129). They play crucial roles in the assembly of immune proteasomes and effective antigen processing (130). The PSME1 overexpression can upregulate the 11S proteasome, thereby activating the hydrolysis of small non-ubiquitinated peptides (131). This enhances the ability of the proteasome to clear misfolded or oxidized proteins and alleviates the oxidative stress response in cardiomyocytes (132). Additionally, overexpression of PSME1 has the potential to reduce myocardial collagen deposition, inhibit cardiomyocyte apoptosis, significantly improve cardiac systolic and diastolic functions, and exert a notable effect on preventing cardiac dysfunction after myocardial infarction reperfusion (133, 134).

Vitamin C, also known as L-ascorbic acid, is not only a cofactor for 15 different enzymes but also a type of acidic hexose derivative (135). It possesses significant reducing properties and effectively enhances the absorption of iron, calcium, and other minerals. Additionally, it plays a crucial role in the synthesis and metabolism of hormones and proteins as well as in epigenetic mechanisms (136). Vitamin C exhibits strong free-radical neutralization capabilities, which significantly mitigates cell damage induced by oxidative stress (137). In addition, they can effectively alleviate inflammatory reactions in blood vessels and promote NO production of nitric oxide to optimize endothelial cell function (138, 139). Vitamin C can reduce angiotensin production, lower blood pressure, regulate blood lipid levels, and improve arterial stiffness (140).

Tyrosylprotein sulfotransferase 2 (TPST2) is a specific integral membrane glycoprotein located in the trans-Golgi network (141). Its primary function is to catalyze the transfer of sulfate from 3’-phosphoadenosine 5’-phosphosulfate (PAPS) to tyrosine residues of proteins (142). Notably, the enhanced activity of TPST2 can trigger atherosclerosis via the aggregation of monocytes and macrophages (143, 144).

Vitamin D plays a crucial role in calcium and phosphorus metabolism and is considered a key hormone precursor that exerts biological effects by activating nuclear vitamin D receptors in myocardial and vascular endothelial cells (145). In recent years, a growing body of research has linked vitamin D, gut microbiota, and the immune system. Vitamin D and the gut microbiota interact in multiple ways to modulate the immune system throughout the body (146). Vitamin D exerts its effects on multiple immune cells via its receptors, thereby modulating cytokine production and inhibiting excessive immune inflammatory responses (147). Vitamin D can influence the differentiation and function of T-cells, promoting the development of more tolerant Treg subpopulations while suppressing the activity of inflammatory effector T-cells, such as Th1, Th2, and Th17 (148). Additionally, specific microbes can induce Treg cell development, fostering anti-inflammatory responses, whereas gut microbiota dysbiosis may elevate levels of pro-inflammatory molecules (149, 150). Notably, vitamin D also contributes to maintaining the intestinal barrier, stabilizing microbiota composition, reducing excessive immune reactions, and inhibiting intestinal inflammation (151). Abnormal activation of the immune system can promote plaque formation and accelerate the progression of atherosclerosis (152). Regulation of vitamin D and microbiota can effectively modulate the immune system, decrease the release of pro-inflammatory cytokines, inhibit vascular wall inflammation, and thus reduce the risk of atherosclerosis. However, the current research on the relationship between vitamin D supplementation and CVD still yields inconsistent conclusions (153–155). Our research suggests that excessive intake of vitamin D may increase the risk of right bundle branch block. A possible reason for this is that excessive vitamin D intake can lead to elevated blood calcium levels, resulting in the deposition of calcium salts and the formation of plaques within the myocardial tissue, thereby affecting myocardial excitability and conductivity (156, 157).

Vitamin E, discovered in the 1920s, is composed of tocopherols and tocotrienols; there are four isomers: α, β, γ, and δ, which are classified based on the number and position of methyl groups attached to the chromanol ring (158). As a well-known antioxidant, vitamin E exhibits significant anti-inflammatory properties, making its potential for the prevention and treatment of CVDs highly anticipated (159). However, despite considerable attention being paid to the role of vitamin E in preventing CVD, its effects remain controversial. However, large-scale clinical trials have not provided conclusive evidence to support its efficacy (160–162). Our study also revealed that vitamin E can significantly reduce the risk of intracerebral hemorrhage and paroxysmal tachycardia but did not provide results regarding the relationship between vitamin E and other CVDs.

### 4.3 Advantages and disadvantages

Our study has several advantages. First, compared to traditional observational studies, MR analysis is more rigorous in exploring causality, effectively avoiding the interference of reverse causality and confounding factors. Second, our exposure and outcome samples were strictly selected from participants of European descent to minimize the impact of population stratification bias, further enhancing the reliability of the study results. In addition, we used the Bayesian–MR method to verify our findings, which enhanced the robustness of our results. Finally, we explored the mediating effect of multiomics on trace element exposure and cardiovascular outcomes to reveal the potential mechanisms underlying this relationship.

Despite our efforts to overcome the numerous limitations of our study, we must acknowledge its constraints. First, when examining the causal relationship between trace elements and CVD, despite controlling for other potential interfering factors as much as possible, we cannot fully exclude the possibility that SNPs associated with trace elements influence CVD through alternative indirect pathways. The potential association of this polymorphism with other traits could introduce confounding effects, thereby posing a challenge to our causal inference. Additionally, even when the F-statistic exceeds 10, the issue of weak instrumental variables persists and the estimated causal effect may still be biased. A larger, more comprehensive prospective cohort study is necessary to further enhance the reliability of our conclusions. Furthermore, the results of this study were primarily based on European population data. While this strategy helps minimize population stratification bias, it also restricts the general applicability of the findings. Genetic and environmental differences may exist among different races and populations, which could lead to heterogeneity in the causal relationships between the two variables across different populations. Finally, although we conducted MR analysis using large-scale genetic data and revealed genetic relationships between trace elements, proteins/serum metabolites, and CVD, we did not conduct additional experimental or observational studies. Further research is needed to validate these findings in the future.

## 5 Conclusion

In summary, our study systematically uncovers the causal relationships between various trace elements and multiple CVDs from a genetic perspective. In addition, we have identified potential mediating effects of specific proteins and serum metabolites in this causal relationship: 1. Potassium can reduce the risk of ischemic heart disease by mediating the expression of proteins C1R and BDH2, as well as modulating serum metabolite 4-methoxyphenol sulfate levels; 2. Vitamin B12 can increase the risk of coronary atherosclerosis by influencing the expression of proteins PSME1 and VPS29; 3. Vitamin B12 can elevate the risk of cardiovascular death through its impact on PSME1 protein expression; 4. Vitamin C, through its regulation of the TPST2 protein, can decrease the risk of cardiac arrest. These findings provide genetic evidence for the role of trace elements in CVD and offer new insights and avenues for the prevention and treatment of CVD.

## Data Availability

The original contributions presented in the study are included in the article/**Supplementary Material**. Further inquiries can be directed to the corresponding authors.
